# A Water-Soluble Leggero Pillar[5]arene

**DOI:** 10.3390/molecules27196259

**Published:** 2022-09-23

**Authors:** Jia-Rui Wu, Gengxin Wu, Zhi Cai, Dongxia Li, Meng-Hao Li, Yan Wang, Ying-Wei Yang

**Affiliations:** International Joint Research Laboratory of Nano-Micro Architecture Chemistry, College of Chemistry, Jilin University, 2699 Qianjin Street, Changchun 130012, China

**Keywords:** leggero pillar[5]arene, host–guest systems, pillararene, molecular recognition

## Abstract

The study of aqueous-phase molecular recognition of artificial receptors is one of the frontiers in supramolecular chemistry since most biochemical processes and reactions take place in an aqueous medium and heavily rely on it. In this work, a water-soluble version of leggero pillar[5]arene bearing eight positively charged pyridinium moieties (**CWP[5]L)** was designed and synthesized, which exhibited good binding affinities with certain aliphatic sulfonate species in aqueous solutions. Significantly, control experiments demonstrate that the guest binding performance of **CWP[5]L** is superior to its counterpart water-soluble macrocyclic receptor in traditional pillararenes.

## 1. Introduction

Synthetic macrocycles have been serving as the primary tools in host-guest chemistry since their birth owing to their intrinsic functional characteristics and capabilities of molecular recognition and self-assembly [1,2,3]. Modern supramolecular chemistry has also significantly benefited from the development of novel macrocyclic receptors with preorganized cavity structures and intriguing host–guest properties [4]. For example, pillar[n]arenes (pillararenes), a family of influential synthetic macrocycles first introduced by Ogoshi et al. in 2008 [5,6], have experienced rapid development during the past years and created a boom in many cross-disciplinary research fields, including but not limited to molecular devices and machines [7], stimuli-responsive supramolecular/host–guest systems [8,9,10,11,12,13,14], porous/nonporous materials [15,16,17], organic–inorganic hybrid systems [18,19,20,21], catalysis [22,23,24] and cancer theranostics [25,26,27].

Inspired by pillararenes, many novel macrocyclic entities such as asar[n]arenes [28], hybrid[n]arenes [29], biphenyl-extended pillar[6]arene [30,31,32,33], tiara[5]arene [34], bowtiearene [35], leaning pillar[6]arene [36,37], geminiarenes [38,39], pagoda[4]arene [40] and biphenarenes [41] have been designed and synthesized in rapid succession, promoting the prosperity of modern synthetic macrocyclic chemistry and creating unlimited possibilities for supramolecular chemistry and materials. Very recently, we presented a new version of pillararene-derived macrocyclic arenes named leggero pillar[n]arenes [42,43], in which two substituents on a single one of the units of traditional pillararenes were selectively removed to expand their structural flexibility and cavity adaptability.

Since most biochemical reactions take place in water, the investigation of molecular recognition in aqueous phases is of greater research value than in organic phases. In the past few years, cationic water-soluble macrocyclic receptors have received tremendous attention in supramolecular community due to their great potential in complexing with important organic anionic species, and many valuable applications such as antibiotics [44], metabolism regulation [45], selective precipitation [33], self-assembly [46] and supra-amphiphiles [47] have been explored on the basis of this feature. Thus, synthesis of novel cationic water-soluble macrocyclic receptors and investigating their host–guest properties in aqueous phase are highly significant and worth exploring.

**Scheme 1:**
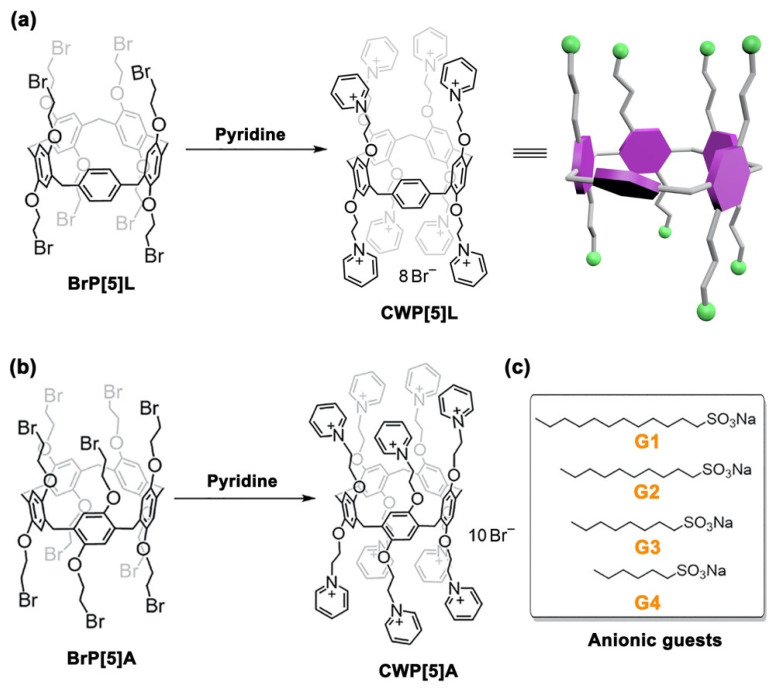
Synthesis of (**a**) **CWP[5]L** and (**b**) **CWP[5]A**. (**c**) Chemical structures of selected aliphatic sulfonate guests **G1**–**G4** investigated in this study.

In this work, the first cationic water-soluble version of the leggero pillar[5]arene (i.e., **CWP[5]L**) is designed and successfully synthesized. Four aliphatic sulfonate guests with different chain lengths (**G1**–**G4**) are selected to investigate the recognition properties of **CWP[5]L** in aqueous media (Scheme 1). Control experiments employing its counterpart macrocyclic derivative **CWP[5]A** from traditional pillararenes confirm that **CWP[5]L** has better binding performance owing to its superior structural flexibility.

## 2. Results

As shown in Scheme 1, **CWP[5]L** and **CWP[5]A** bearing eight and ten cationic pyridinium moieties could be quantitatively prepared through a S_N_2 nucleophilic substitution by reacting their corresponding perbromoethylated macrocyclic derivatives BrP[5]L and BrP[5]A with pyridine (also as solvent), respectively, and the target receptors were fully characterized by ^1^H/^13^C NMR and high-resolution mass spectroscopy (HRMS) (Figures S1–S6).

**Figure 1:**
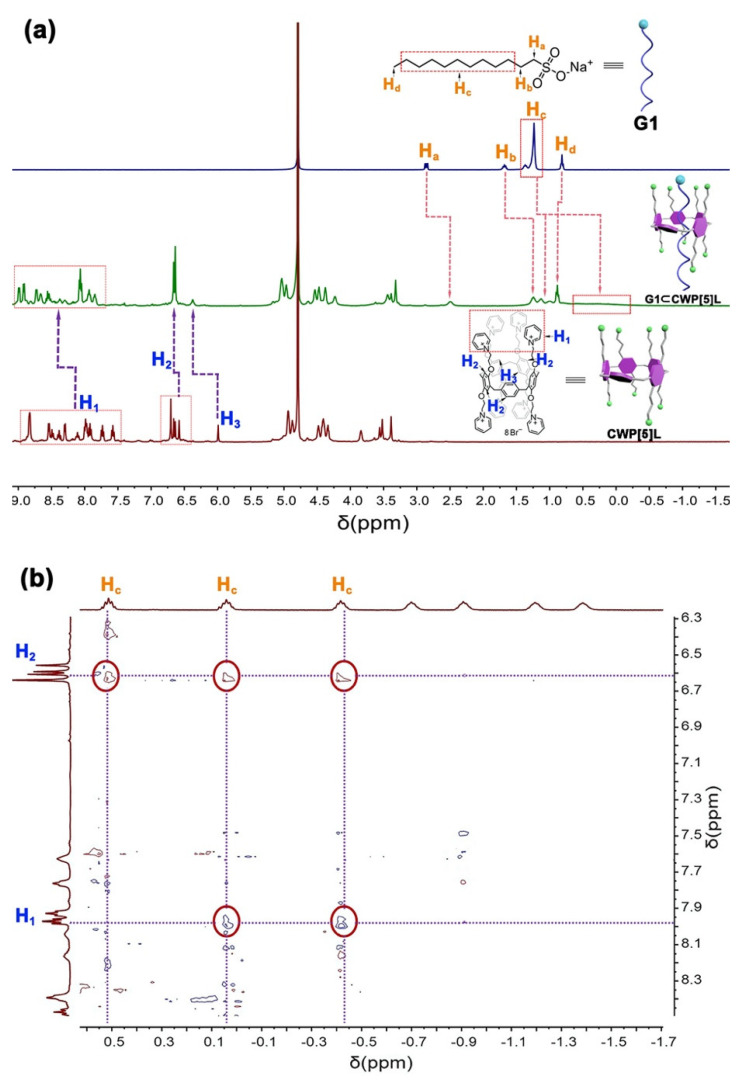
(**a**) ^1^H NMR spectra (400 MHz, D_2_O, 298 K) of **G1**, **G1** + **CWP[5]L** and **CWP[5]L**. Concentrations of host and guest are all 4.0 mM; (**b**) 2D ROE analysis of **CWP[5]L** with **G1** in D_2_O with a mixing time of 300 ms (600 MHz, 298 K; the concentrations of **CWP[5]L** and **G1** are 4 mM and 5 mM, respectively).

The binding behaviors of **CWP[5]L** with **G1**–**G4** were first investigated by ^1^H NMR spectroscopy. As shown in Figure 1a, when we mixed **CWP[5]L** (4.0 mM) and 1.0 equiv. of **G1** in D_2_O, the ^1^H NMR spectrum displayed only one set of resonance signals distinct from those of the single component host and guest, indicating that the binding complex was formed, and the complexation between **CWP[5]L** and **G1** was a fast exchange process on the NMR timescale. Compared with the free guest, protons H_a_-H_c_ of **G1** showed remarkable upfield shifts and broadening effects as a result of the inclusion-induced shielding effects, while protons H_d_ on the terminal methyl showed downfield shifts due to the deshielding effect. These changes indicated that **G1** was threaded into the host cavity, forming an interpenetrated inclusion complex. On the other hand, the host was deshielded by the included guest, and the proton signals of **CWP[5]L** derived from the pyridinium moieties (H_1_) and substituted phenylene subunits (H_2_ and H_3_) exhibited downfield displacement.

**Figure 2:**
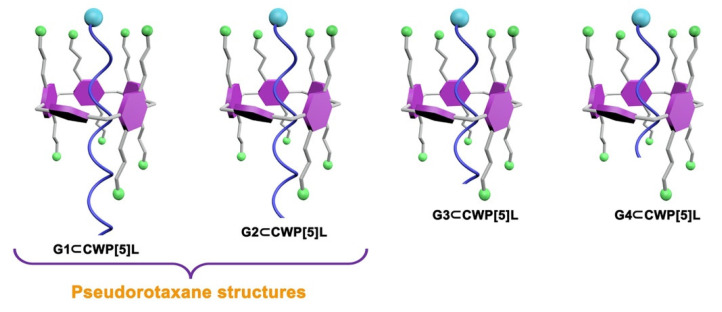
Cartoon representation of the 1:1 inclusion complexation between **CWP[5]L** and guests **G1**–**G4** (from left to right), and the [2]pseudorotaxane structures of the complexes of **G1**⊂**CWP[5]L** and **G2**⊂**CWP[5]L**.

The host-guest binding behavior between **CWP[5]L** and **G1** was also investigated by 2D ROESY analysis. As shown in Figure 1b, the correlation signals between the alkyl chain protons (H_c_) of **G1** and the phenylene and pyridinium protons (H_1_ and H_2_) of **CWP[5]L** were clearly observed, further confirming the interpenetrated geometry. Besides, similar complexation-induced shielding/deshielding effects were also observed in the mixtures of **CWP[5]L** and other selected sulfonate guests (**G2**–**G4**), respectively, indicating that these host-guest assemblies have a similar binding mode, i.e., the macrocyclic cavity of **CWP[5]L** is threaded by the alkyl chain of the guests. Interestingly, it should be noted that with the decrease in the alkyl chain length from **G1** to **G4**, the resonance signals for the terminal methyl protons (H_d_) experienced a shift from downfield (**G1** and **G2**) to upfield (**G3** and **G4**) upon mixing with **CWP[5]L** (Figure 1a and Figures S7–S9), suggesting that the terminal groups of **G1** and **G2** are protruded out of the macrocyclic cavity and the corresponding inclusion complexes could also be considered as [2]pseudorotaxane structures, respectively (Figure 2).

**Figure 3:**
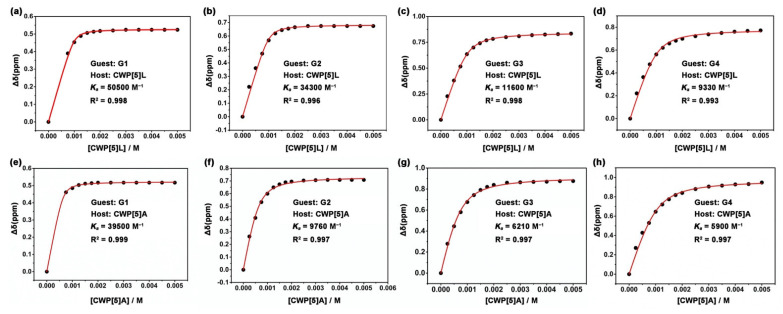
Non-linear curve-fitting (NMR titrations) for the complexation between (**a**) **CWP[5]L** and G1, (**b**) **CWP[5]L** and G2, (**c**) **CWP[5]L** and G3, (**d**) **CWP[5]L** and G4, (**e**) **CWP[5]A** and G1, (**f**) **CWP[5]A** and G2, (**g**) **CWP[5]A** and G, and (**h**) **CWP[5]A** and G4 in D_2_O at 298 K. All the titration experiments were repeated thrice with the relative errors less than 15%.

To quantitatively estimate the interactions between **CWP[5]L** and the sulfonate guests, ^1^H NMR titration experiments were further implemented to afford the association constants (*K*_a_) for the host-guest inclusion complexation in D_2_O (Figures S10–S13). As shown in Figure 3a–d, **CWP[5]L** has good binding affinities towards all the guests, and the *K*_a_ values for 1:1 complexation with **G1**–**G4** were determined to be 50,500, 34,300, 11,600 and 9330 M^−1^, respectively. Interestingly, a marked increase in the *K*_a_ values was observed in the order of **G4** < **G3** < **G2** < **G1**, which could be attributed to the enhanced hydrophobic interaction and the guests’ increasing chain length.

Given that **CWP[5]L** exhibited strong binding affinities with linear sulfonate guests, we were curious to find some differences in the binding performance between **CWP[5]L** and its counterpart water-soluble macrocyclic derivative **CWP[5]A** from traditional pillararenes. Subsequently, controlled ^1^H NMR titration experiments by using **CWP[5]A** were carried out (Figures S14–S17), and the *K*_a_ values for **G1**–**G4** were determined to be 39,500, 9760, 6210 and 5900 M^−1^, respectively (Figure 3e–h). Analogously, the *K*_a_ values increase in the order of **G4** < **G3** < **G2** < **G1**, again confirming the pivotal role of hydrophobic interaction in modulating the host-guest inclusion complexation in the aqueous phase. Significantly, the binding constants of **CWP[5]L** are larger than those of its counterpart **CWP[5]A**, indicating that the de-functionalized and free-rotation phenylene units invest **CWP[5]L** with more superior cavity adaptability and relatively low in-cavity electron density for binding anionic species.

## 3. Materials and Methods

### 3.1. General Information

Starting reagents and solvents were commercially available and used without further purification unless stated otherwise. ^1^H NMR and ^13^C NMR spectra were recorded on a Bruker AVANCE III 400 MHz instrument at room temperature. The 2D ROESY NMR spectra were recorded on a Bruker AVANCE III 600 MHz instrument. Mass spectra were recorded on a Bruker Agilent1290-micrOTOF Q II High-resolution (HR) mass spectrometry instrument.

### 3.2. Synthetic Procedures

Perbromoethylated macrocyclic derivatives BrP[5]L and BrP[5]A were synthesized according to the previous literature reports [21,43].

#### 3.2.1. Synthesis of **CWP[5]L**

A pyridine (3 mL) solution of BrP[5]L (0.6 g, 0.4 mmol) was heated at 100 °C for 10 h. Then, the resulting precipitate was filtered and washed with CH_2_Cl_2_ to afford the target compound as a light yellow solid (0.82 g, 95%).

^1^H NMR (600 MHz, 298 K, D_2_O, δ ppm): δ 8.88 (dd, *J* = 5.2, 3.5 Hz, 8H), 8.63–8.52 (m, 6H), 8.47–8.35 (m, 6H), 8.17 (t, *J* = 7.9 Hz, 2H), 8.01 (dt, *J* = 40.1, 7.2 Hz, 10H), 7.71 (dt, *J* = 95.8, 7.2 Hz, 8H), 6.77–6.61 (m, 10H), 6.06 (s, 2H), 5.05–4.87 (m, 13H), 4.57–4.52 (m, 4H), 4.50–4.44 (m, 8H), 4.42–4.37 (m, 4H), 3.91 (s, 3H), 3.59 (d, *J* = 22.0 Hz, 6H), 3.44 (s, 4H); ^13^C NMR (101 MHz, 298 K, D_2_O, δ ppm): δ 149.40, 146.35, 146.16, 145.74, 144.81, 144.68, 144.46, 128.30, 128.23, 128.12, 127.70, 116.25, 115.36, 67.44, 66.80, 61.00, 60.90, 34.52, 28.88; HRMS (ESI): [C_91_H_93_N_8_O_8_Br_5_]^3+^ calcd. [M] m/z: 607.1032, found m/z: 607.1082.

#### 3.2.2. Synthesis of **CWP[5]A**

A pyridine (3 mL) solution of BrP[5]A (0.6 g, 0.35 mmol) was heated at 100 °C for 10 h. Then, the resulting precipitate was filtered and washed with CH_2_Cl_2_ to afford the target compound as a light yellow solid (0.84 g, 96%).

^1^H NMR (400 MHz, 298 K, D_2_O, δ ppm): δ 8.69 (d, J = 5.8 Hz, 20H), 8.26 (t, J = 7.8 Hz, 10H), 7.84 (t, J = 7.0 Hz, 20H), 6.48 (s, 10H), 4.88 (s, 20H), 4.42 (s, 20H), 3.37 (s, 10H); ^13^C NMR (101 MHz, 298 K, D_2_O, δ ppm): δ 149.08, 146.08, 144.64, 128.94, 128.05, 115.77, 67.14, 60.82, 29.26; HRMS (ESI): [C_105_H_110_N_10_O_10_Br_6_]^4+^ calcd. [M] m/z: 537.8703, found m/z: 537.8414.

### 3.3. Determination of Association Constants

To determine the association constant, ^1^H NMR titrations using a nonlinear least-squares curve-fitting method were performed at 298 K in D_2_O. The association constants (*K*_a_) were obtained for each host-guest combination from the following equation [48]:

Δδ = (Δδ_∞_/[G]_0_) (0.5[H]_0_ + 0.5([G]_0_ + 1/*K*_a_) − (0.5([H]_0_^2^ + (2[H]_0_(1/*K*_a_ − [G]_0_)) + (1/*K*_a_ + [G]_0_)^2^)^0.5^))

where [H]_0_ is the varying concentrations of host, Δδ is the chemical shift change of specific proton (H_a_) on the guest at [H]_0_, Δδ_∞_ is the chemical shift change of H_a_ when the guest is completely complexed, [G]_0_ is the fixed initial concentration of the guest. Assuming 1: 1 binding mode between the macrocyclic hosts (**CWP[5]L** and **CWP[5]A**) and sulfonate guests **G1**–**G4**, the plot of Δδ as a function of [H]_0_ for each investigated host-guest pair gave a good fit, confirming the validity of the 1:1 complexation stoichiometry assumed.

## 4. Conclusions

In conclusion, we successfully synthesized the first water-soluble derivative of leggero pillar[5]arene (i.e., **CWP[5]L**), where the presence of eight cationic pyridinium moieties makes it a highly effective receptor for anionic sulfonate species in aqueous solutions. NMR experiments demonstrated that all the guests could be encapsulated into the cavity of **CWP[5]L** to form 1:1 host-guest complexes with interpenetrated geometry, and the binding affinities could be remarkably enhanced by increasing the alkyl chain length of the guests due to the growing hydrophobic interaction. More importantly, controlled experiments employing its counterpart water-soluble macrocyclic receptor derived from traditional pillararenes confirmed that **CWP[5]L** had better binding performance because of its superior structural flexibility and cavity adaptability originating from the free-rotation phenylene subunit. Above all, given the superior binding performance toward anionic species in water, we firmly believe that **CWP[5]L** will find far-ranging applications in, for example, molecular recognition, environmental remediation, biomedicine, material science and other related fields.

## Data Availability

Not applicable.

## Supplementary Materials

The following supporting information can be downloaded at: https://www.mdpi.com/article/10.3390/molecules27196259/s1, Figures S1–S6: Characterization of **CWP[5]L** and **CWP[5]A**; Figures S7–S17: Host-guest binding studies by ^1^H NMR spectra.

## Sample Availability

Samples of the compounds are available from the authors.
