# Supplementary material for: A Water-Soluble Leggero Pillar[5]arene
Source: Molecules. 2022 Sep 23;27(19):6259. doi: 10.3390/molecules27196259 (PMC9571795; doi:10.3390/molecules27196259)
Supplement: Supplementary file 1 [file molecules-27-06259-s001.zip › molecules-1929577-supplementary.pdf]

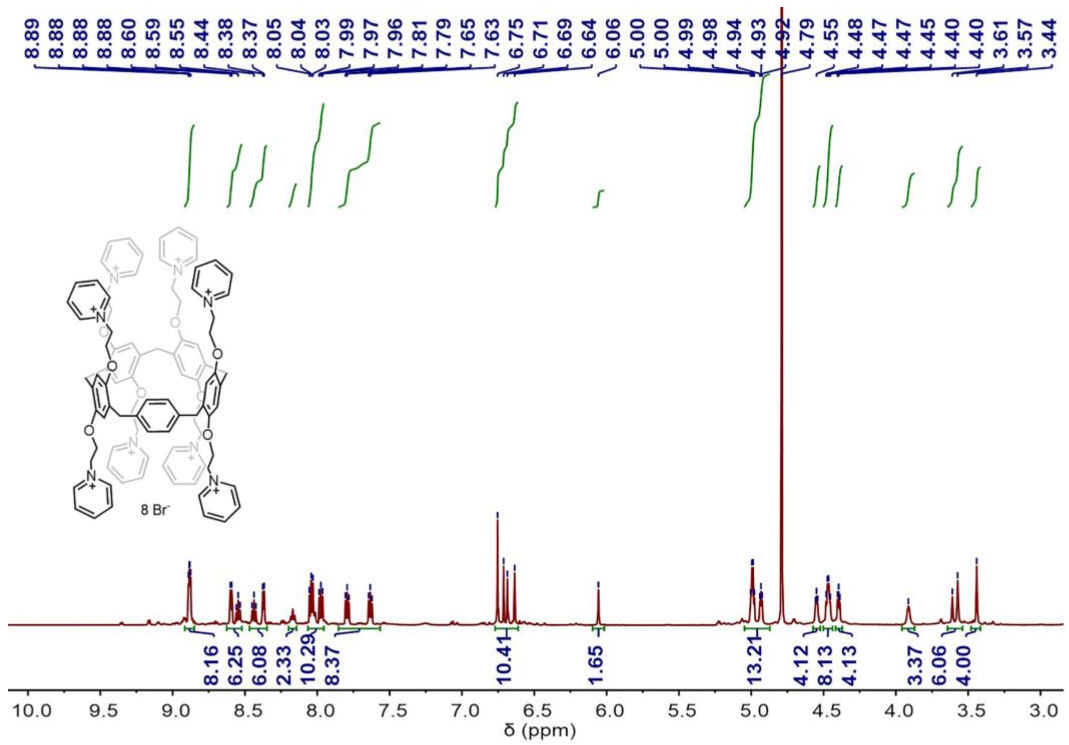

Figure S1. ^{1}H NMR spectrum (600 MHz, 298 K, D_2O) of **CWP[5]L**.

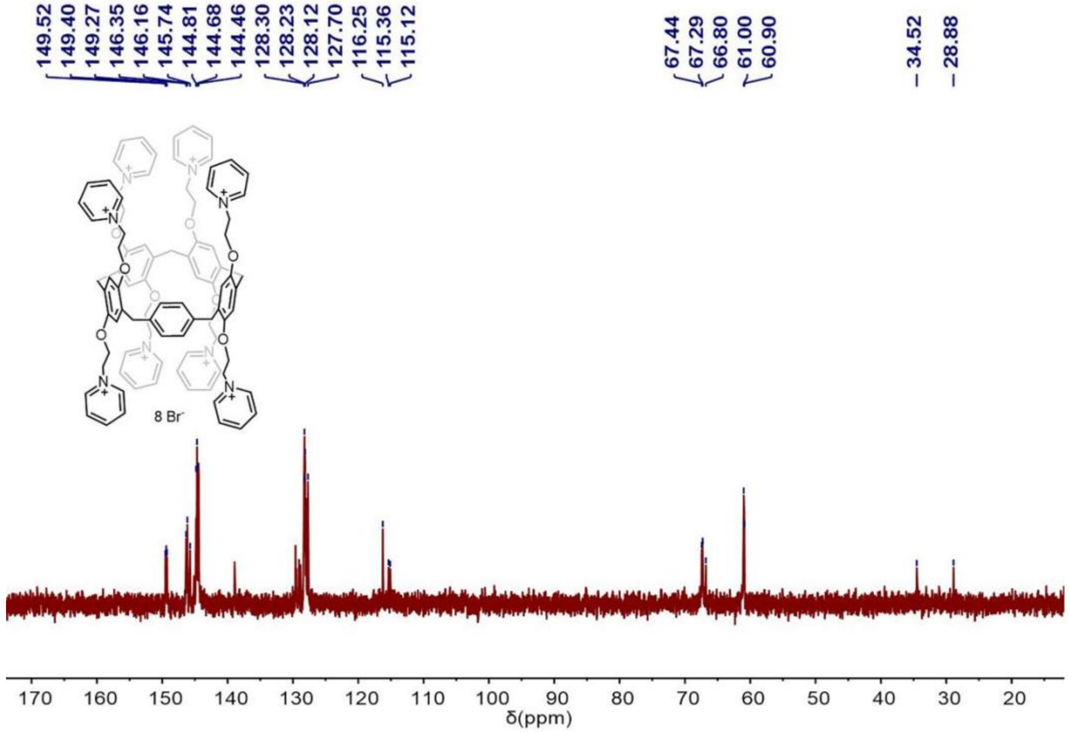

Figure S2. ^{13}C NMR spectrum (101 MHz, 298 K, D_2O) of **CWP[5]L**.

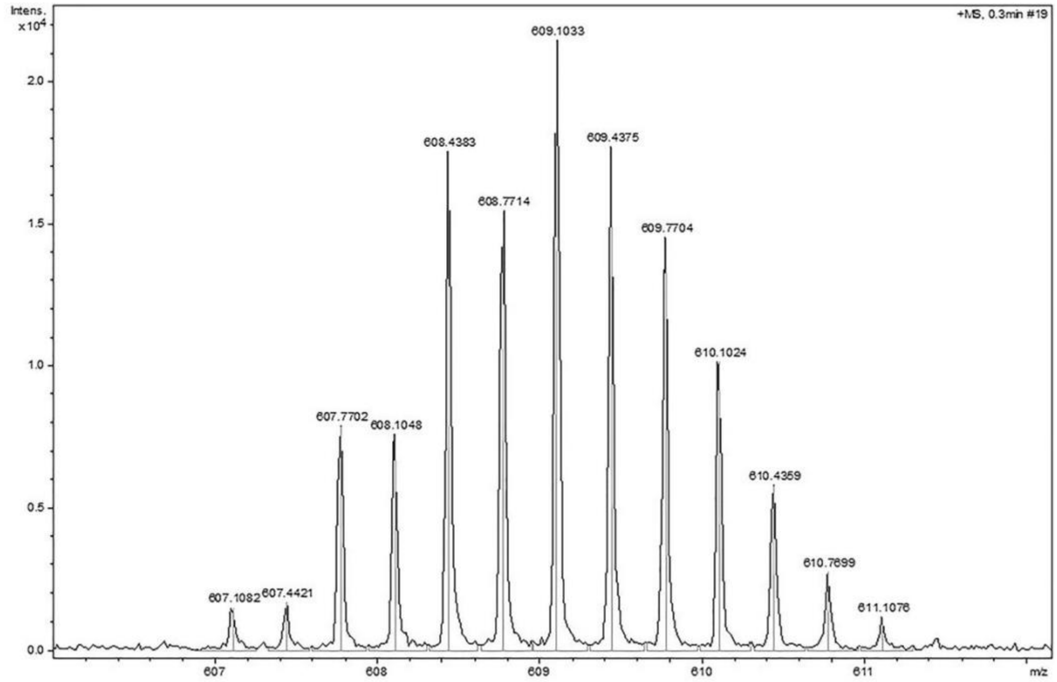

Figure S3. HRMS (ESI) spectrum of **CWP[5]L**.

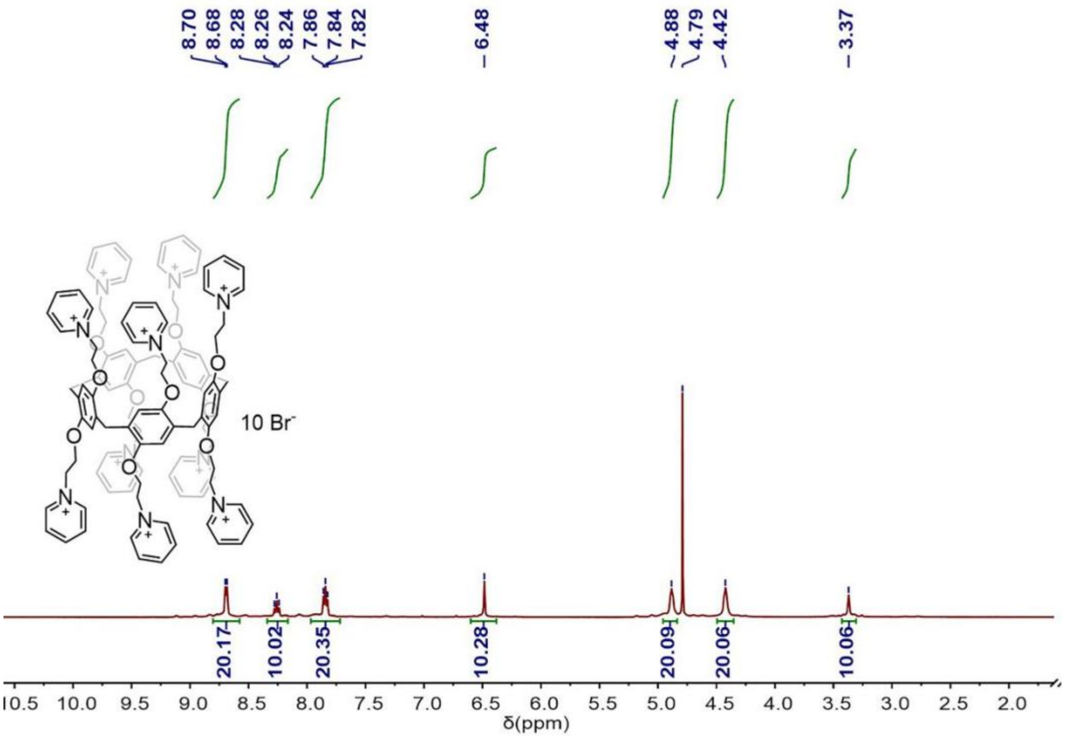

Figure S4. ^{1}H NMR spectrum (400 MHz, 298 K, D_2O) of **CWP[5]A**.

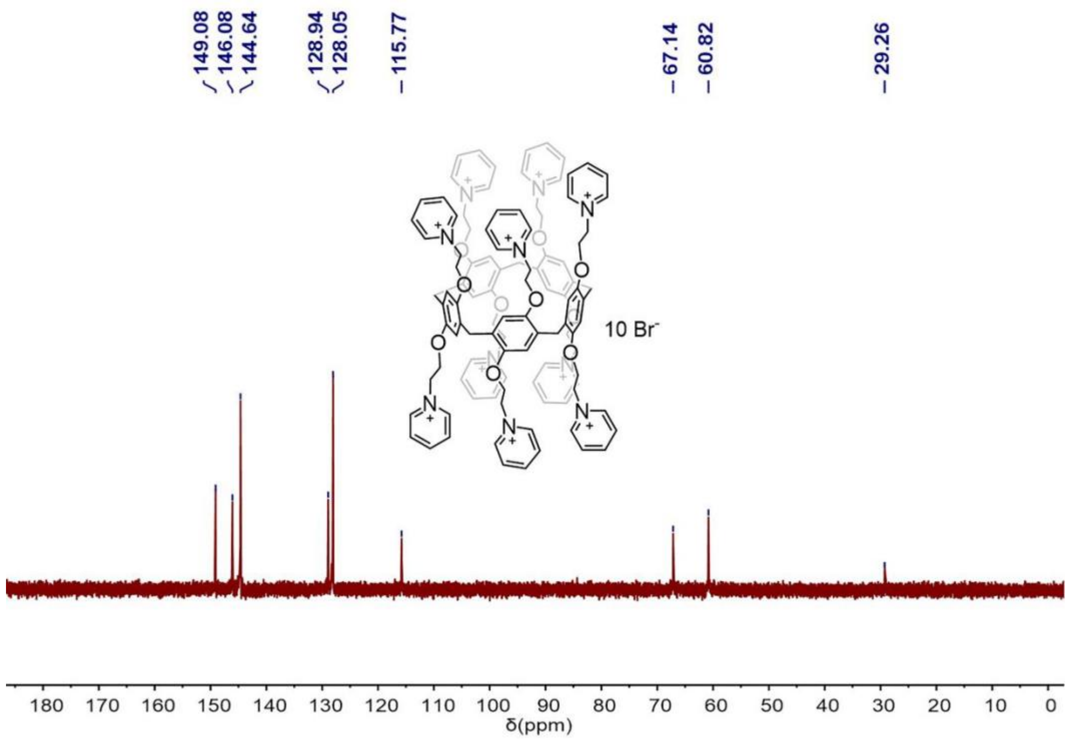

Figure S5. ^{13}C NMR spectrum (101 MHz, 298 K, D_2O) of **CWP[5]A**.

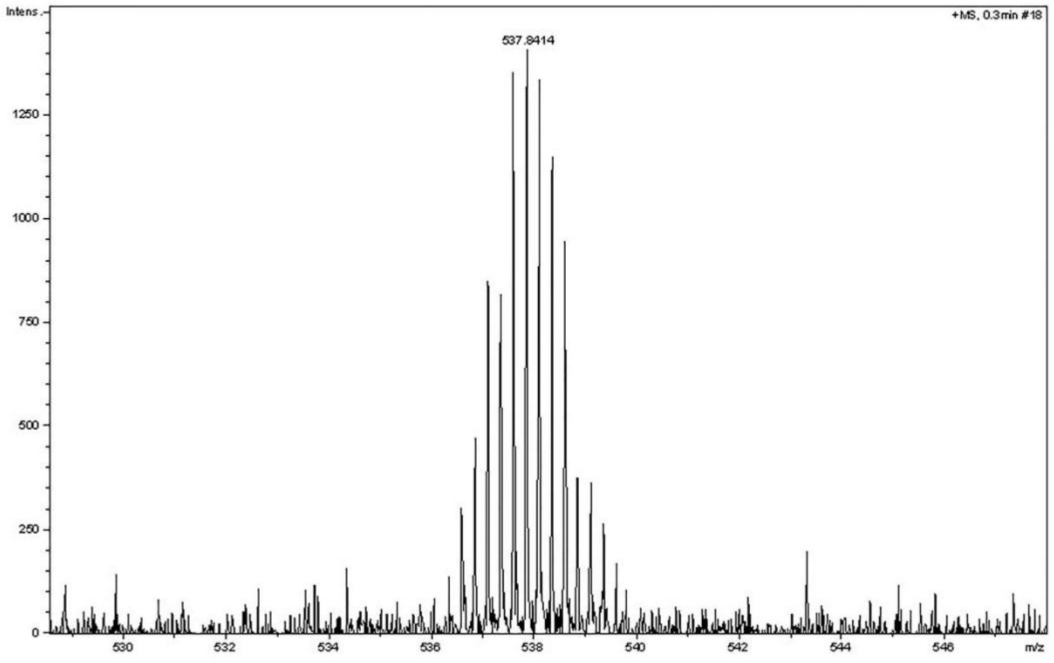

Figure S6. HRMS (ESI) spectrum of **CWP[5]A**.

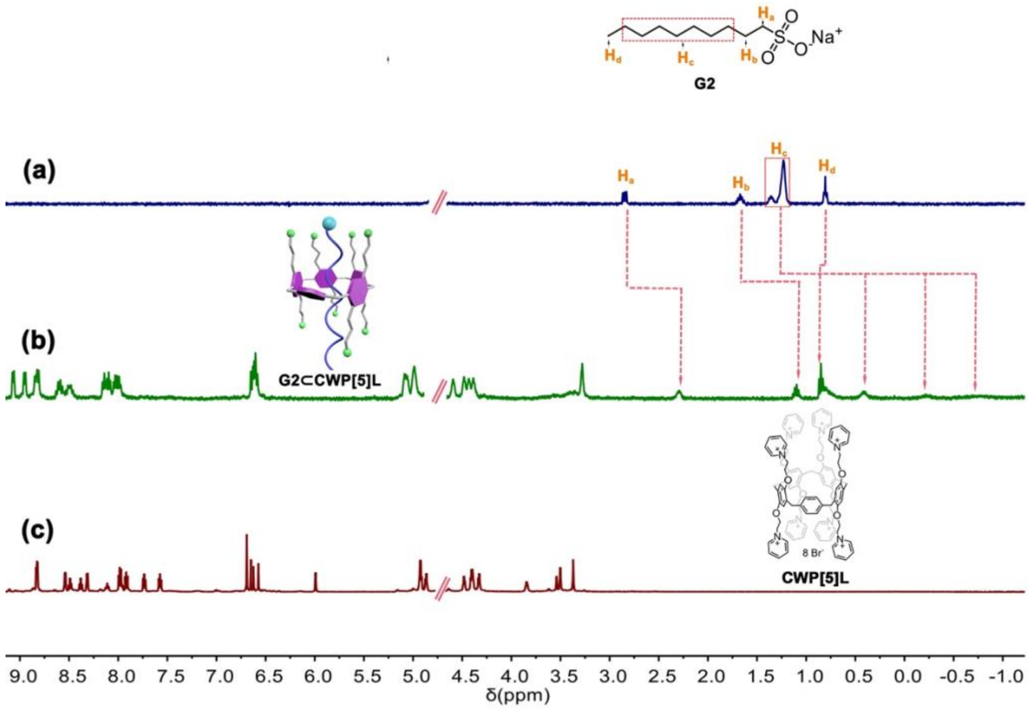

Figure S7. (a) ^{1}H NMR spectra (400 MHz, 298 K, D_2O) of (a) **G2**, (b) **CWP[5]L** + **G2** and (c) **CWP[5]L**. Concentrations of host and guest are all 4.0 mm.

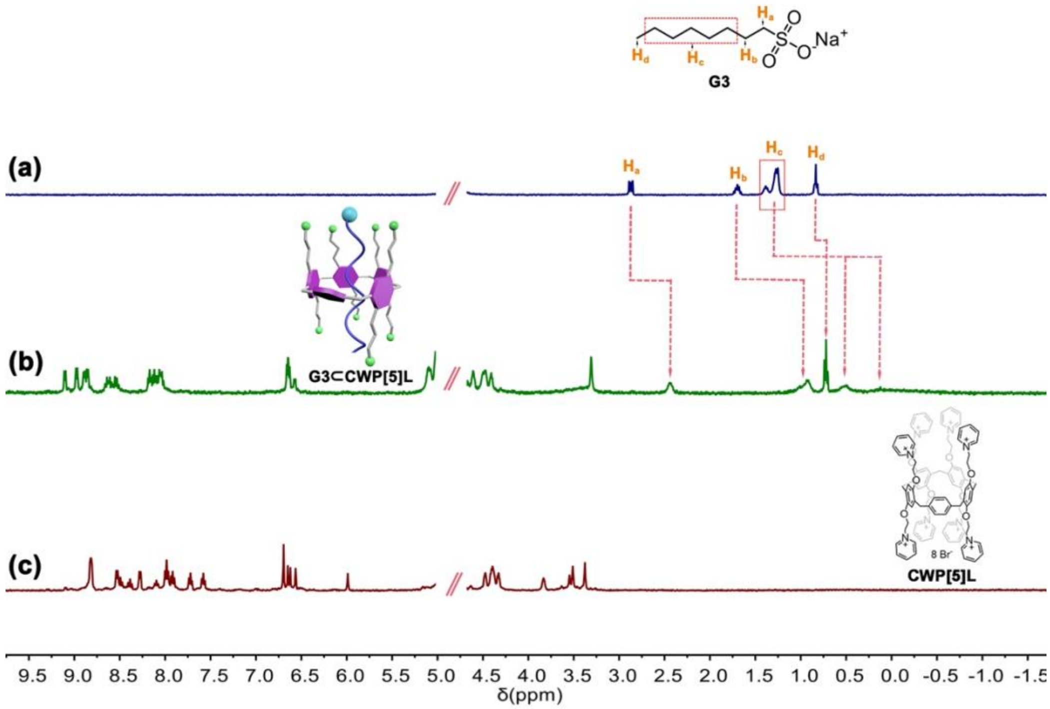

Figure S8. (a) ^{1}H NMR spectra (400 MHz, 298 K, D_2O) of (a) **G3**, (b) **CWP[5]L** + **G3** and (c) **CWP[5]L**. Concentrations of host and guest are all 4.0 mm.

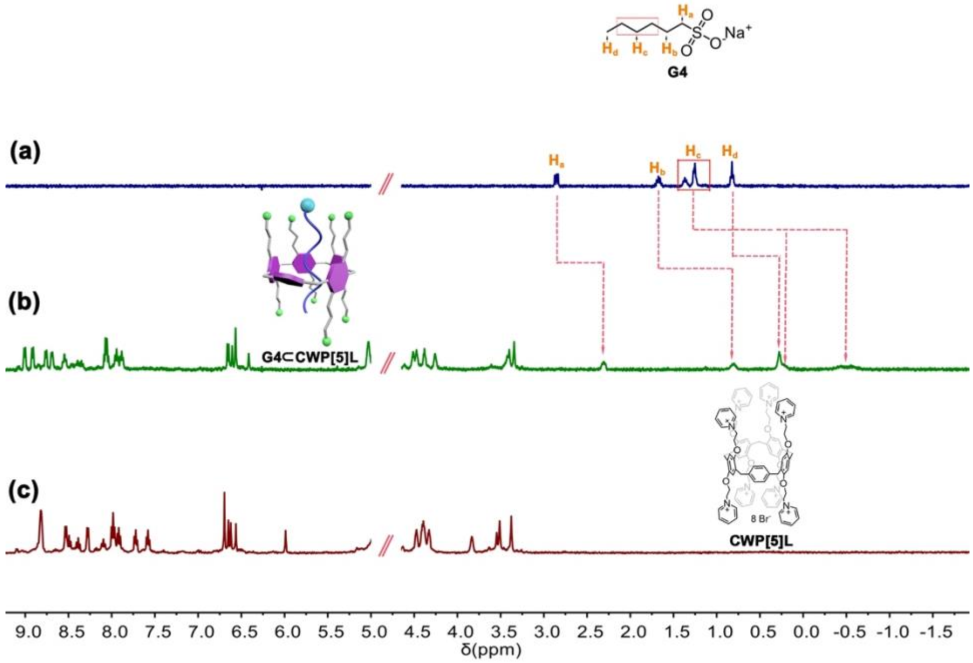

Figure S9. (a) ¹H NMR spectra (400 MHz, 298 K, D_2O) of (a) **G4**, (b) **CWP[5]L** + **G4** and (c) **CWP[5]L**. Concentrations of host and guest are all 4.0 mm.

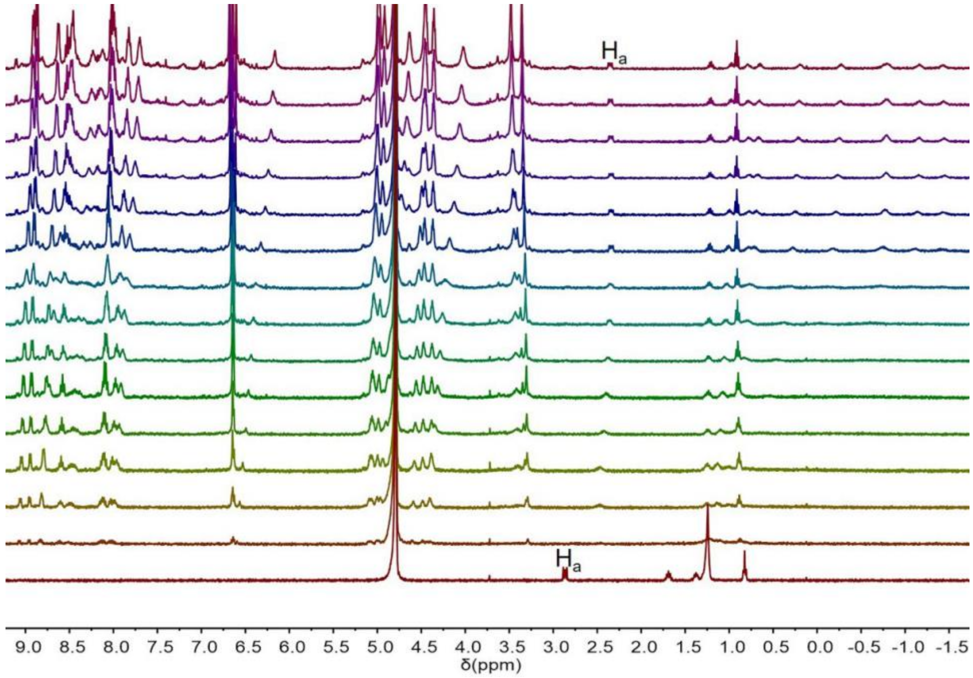

Figure S10. Partial ¹H NMR spectra (400 MHz, D_2O, 293 K) of **G1** at a concentration of 1 mM upon titration of **CWP[5]L**. From bottom to top, the concentration of **CWP[5]L** was 0, 0.25, 0.50, 0.75, 1.00, 1.25, 1.50, 1.75, 2.00, 2.50, 3.00, 3.50, 4.00, 4.50 and 5.00 mM, respectively.

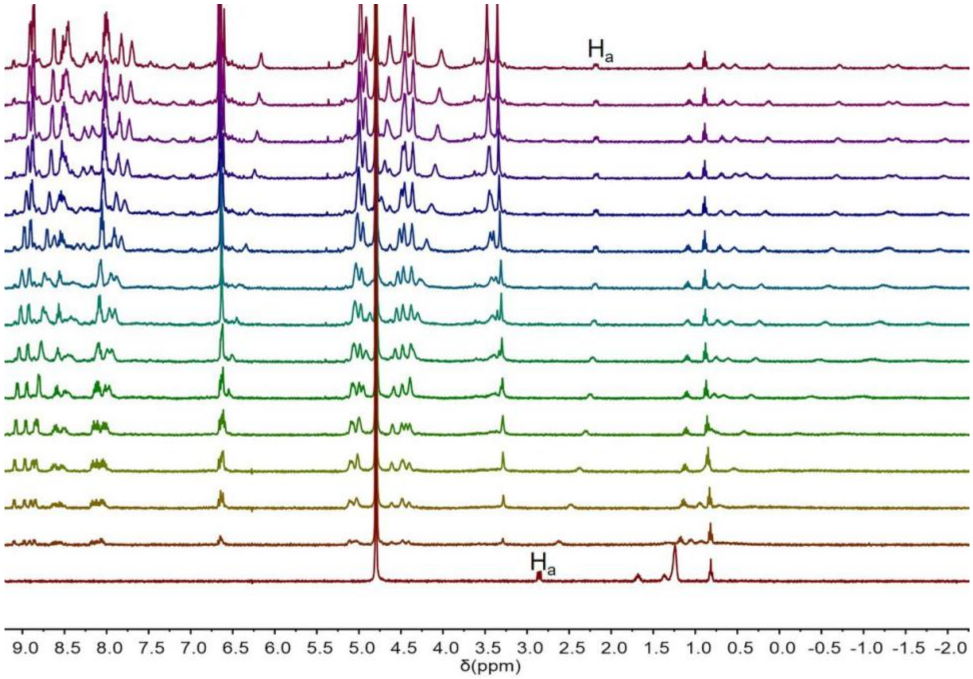

Figure S11. Partial ^{1}H NMR spectra (400 MHz, D_2O, 293 K) of **G2** at a concentration of 1 mM upon titration of **CWP[5]L**. From bottom to top, the concentration of **CWP[5]L** was 0, 0.25, 0.50, 0.75, 1.00, 1.25, 1.50, 1.75, 2.00, 2.50, 3.00, 3.50, 4.00, 4.50 and 5.00 mM, respectively.

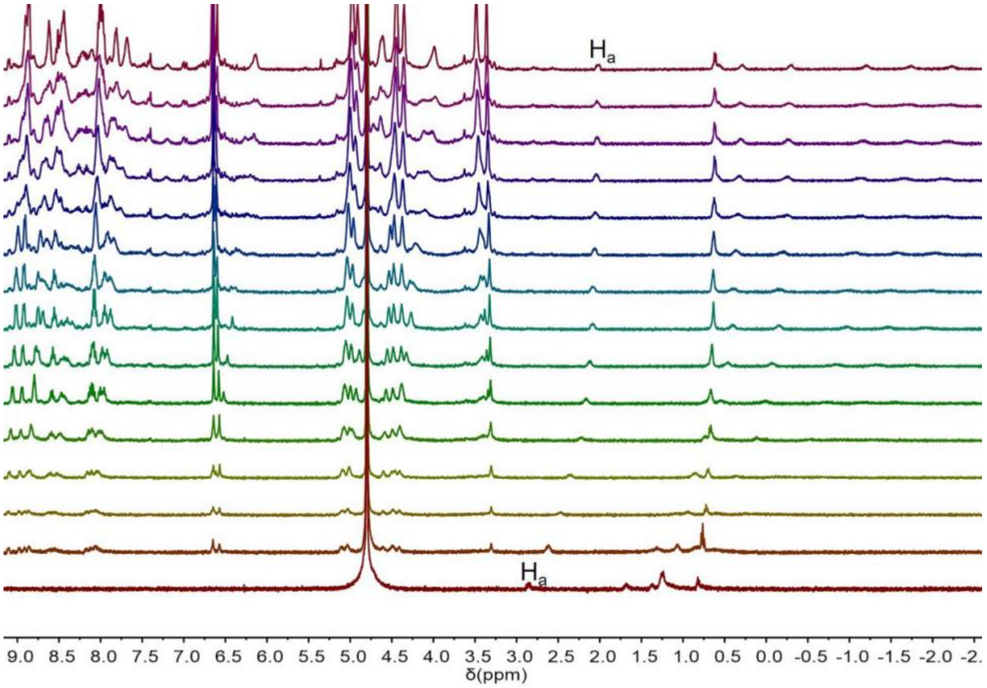

Figure S12. Partial ^{1}H NMR spectra (400 MHz, D_2O, 293 K) of **G3** at a concentration of 1 mM upon titration of **CWP[5]L**. From bottom to top, the concentration of **CWP[5]L** was 0, 0.25, 0.50, 0.75, 1.00, 1.25, 1.50, 1.75, 2.00, 2.50, 3.00, 3.50, 4.00, 4.50 and 5.00 mM, respectively.

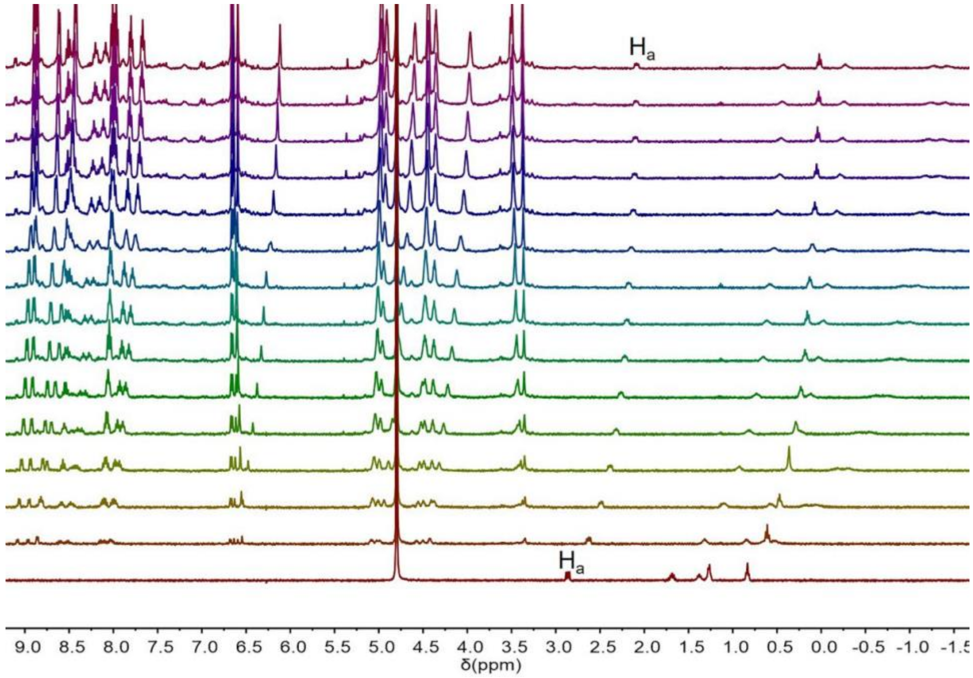

Figure S13. Partial ^{1}H NMR spectra (400 MHz, D_2O, 293 K) of **G4** at a concentration of 1 mM upon titration of **CWP[5]L**. From bottom to top, the concentration of **CWP[5]L** was 0, 0.25, 0.50, 0.75, 1.00, 1.25, 1.50, 1.75, 2.00, 2.50, 3.00, 3.50, 4.00, 4.50 and 5.00 mM, respectively.

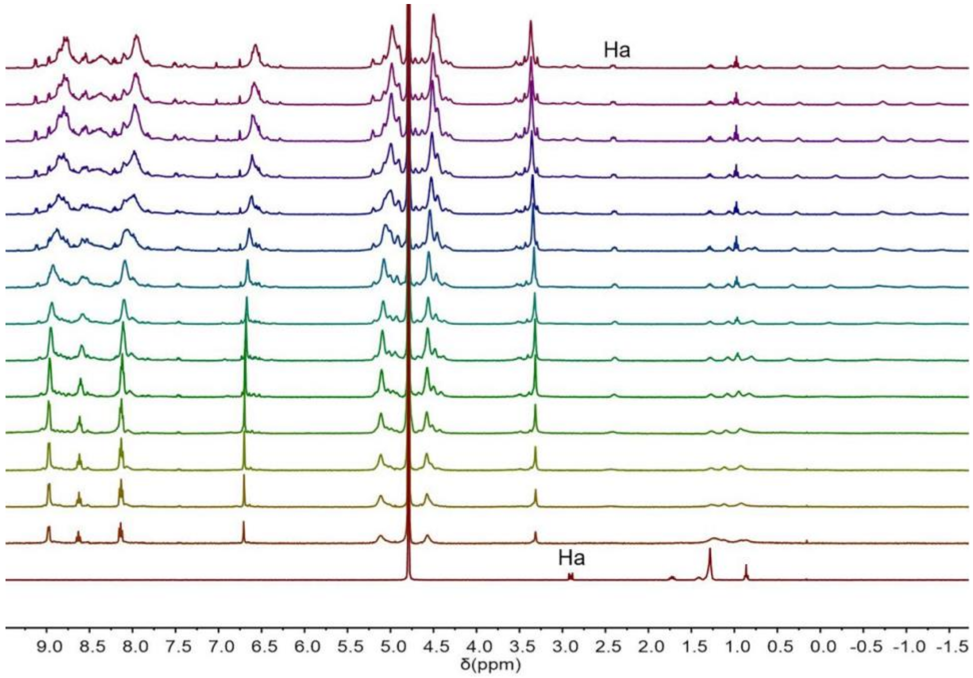

Figure S14. Partial ^{1}H NMR spectra (400 MHz, D_2O, 293 K) of **G1** at a concentration of 1 mM upon titration of **CWP[5]A**. From bottom to top, the concentration of **CWP[5]A** was 0, 0.25, 0.50, 0.75, 1.00, 1.25, 1.50, 1.75, 2.00, 2.50, 3.00, 3.50, 4.00, 4.50 and 5.00 mM, respectively.

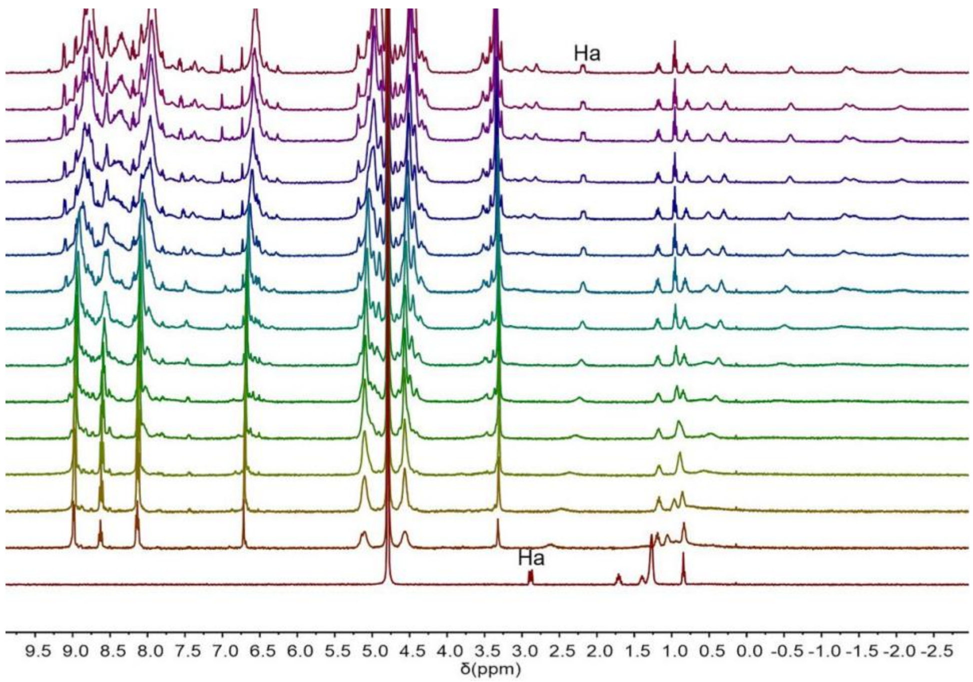

Figure S15. Partial ^{1}H NMR spectra (400 MHz, D_2O, 293 K) of **G2** at a concentration of 1 mM upon titration of **CWP[5]A**. From bottom to top, the concentration of **CWP[5]A** was 0, 0.25, 0.50, 0.75, 1.00, 1.25, 1.50, 1.75, 2.00, 2.50, 3.00, 3.50, 4.00, 4.50 and 5.00 mM, respectively.

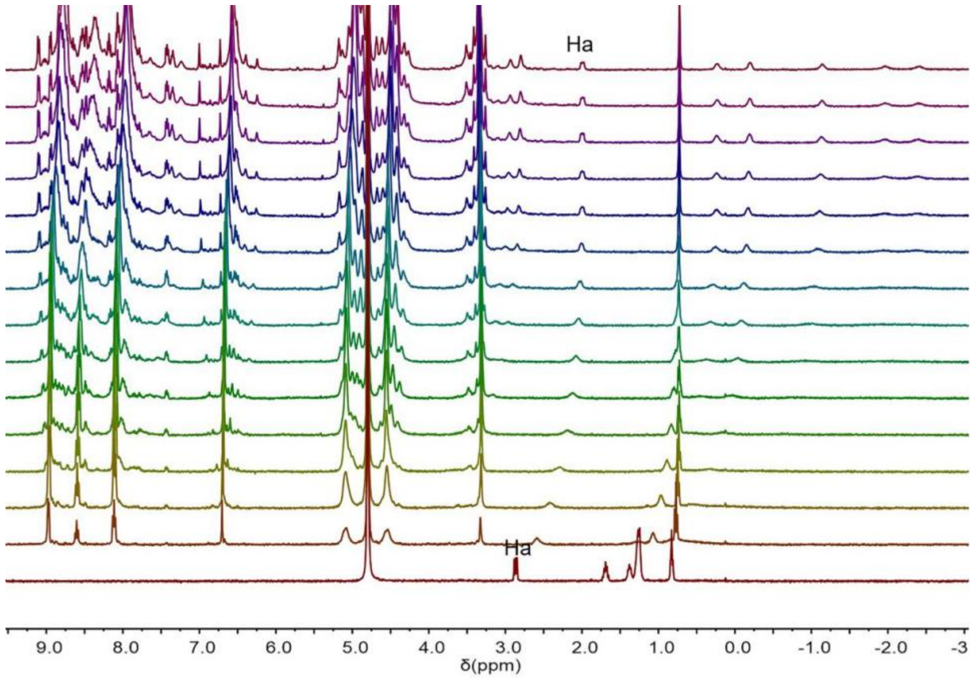

Figure S16. Partial ^{1}H NMR spectra (400 MHz, D_2O, 293 K) of **G3** at a concentration of 1 mM upon titration of **CWP[5]A**. From bottom to top, the concentration of **CWP[5]A** was 0, 0.25, 0.50, 0.75, 1.00, 1.25, 1.50, 1.75, 2.00, 2.50, 3.00, 3.50, 4.00, 4.50 and 5.00 mM, respectively.

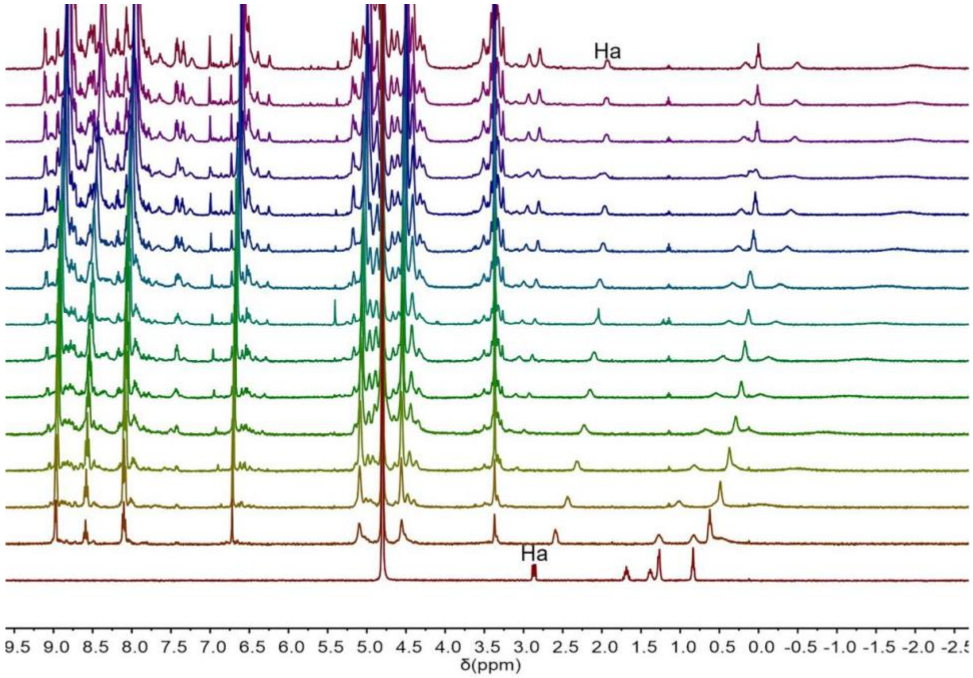

Figure S17. Partial ^{1}H NMR spectra (400 MHz, D_2O, 293 K) of **G4** at a concentration of 1 mM upon titration of **CWP[5]A**. From bottom to top, the concentration of **CWP[5]A** was 0, 0.25, 0.50, 0.75, 1.00, 1.25, 1.50, 1.75, 2.00, 2.50, 3.00, 3.50, 4.00, 4.50 and 5.00 mM, respectively.
